# Analysis of Pole-Ascending–Descending Action by Insects Subjected to High Voltage Electric Fields

**DOI:** 10.3390/insects11030187

**Published:** 2020-03-16

**Authors:** Yoshinori Matsuda, Yoshihiro Takikawa, Koji Kakutani, Teruo Nonomura, Hideyoshi Toyoda

**Affiliations:** 1 Faculty of Agriculture, Kindai University, Nara 631-8505, Japan; nonomura@nara.kindai.ac.jp; 2 Plant Center, Institute of Advanced Technology, Kindai University, Wakayama 642-0017, Japan; takikawa@waka.kindai.ac.jp; 3 Pharmaceutical Research and Technology Institute, Kindai University, Osaka 577-8502, Japan; kakutani@kindai.ac.jp; 4 Anti-Aging Centers, Kindai University, Osaka 577-8502, Japan; 5 Research Association of Electric Field Screen Supporters, Nara 631-8505, Japan; toyoda@nara.kindai.ac.jp

**Keywords:** rice weevil, cigarette beetle, avoidance behavior, electric field

## Abstract

The present study was conducted to establish an electrostatic-based experimental system to enable new investigations of insect behavior. The instrument consists of an insulated conducting copper ring (ICR) linked to a direct current voltage generator to supply a negative charge to an ICR and a grounded aluminum pole (AP) passed vertically through the center of the horizontal ICR. An electric field was formed between the ICR and the AP. Rice weevil (*Sitophilus oryzae*) was selected as a model insect due to its habit of climbing erect poles. The electric field produced a force that could be imposed on the insect. In fact, the negative electricity (free electrons) was forced out of the insect to polarize its body positively. Eventually, the insect was attracted to the oppositely charged ICR. The force became weaker on the lower regions of the pole; the insects sensed the weaker force with their antennae, quickly stopped climbing, and retraced their steps. These behaviors led to a pole-ascending–descending action by the insect, which was highly reproducible and precisely corresponded to the changed expansion of the electric field. Other pole-climbing insects including the cigarette beetle (*Lasioderma serricorne*), which was shown to adopt the same behavior.

## 1. Introduction

Electrostatic devices have been developed as physical tools to create pest-free spaces in various facilities based on their insect-capturing [1,2,3,4] and -repelling functions [5]. The insect-capturing function is due to an attractive force produced in the electric field that traps insects when they enter the field. This function was verified using major insect pests in a greenhouse (whiteflies, western flower thrips, green peach aphid, tomato leaf-minor flies, green rice leaf hopper, and shore flies), a warehouse (cigarette beetles, rice weevils, red flour beetles, Azuki been weevils, vinegar flies), a museum (book lice), and a domestic house (common clothes moth, bathroom flies, German cockroach, Oriental termites, and Asian tiger mosquitoes) [6].

The insect-repelling function of the electrostatic apparatus depends on the intrinsic behavior of insects in response to an electric field; that is, insects are deterred from entering the electric field of the apparatus. This characteristic behavior has been confirmed in a wide range of pest and non-pest insects (17 orders, 42 families, 45 genera, and 82 insect species) [5]. These results suggest that insects have a natural tendency to avoid electric fields. However, because insects are not exposed to electric fields under natural conditions, it remains unclear how they were able to sense their existence.

To investigate this phenomenon further, we aimed to construct an experimental that enabled ethological analysis of insects that avoided electric fields and to select the model insects suitable for this purpose. An insect-climbing assay was selected as the most effective experimental method [5,7], and suitable model insects included those with the habit of climbing an upright pole. In the present study, rice weevil and cigarette beetle were selected as model insects because adults of these species are known to climb a straight pole at a constant pace based on routine observation of many insect species. These pests can multiply rapidly in storage bags and spoil postharvest crops; therefore, they have been targeted as serious pests in postharvest crop protection [8]. Cigarette beetle adults damage a wide range of stored agricultural products, including cocoa, beans, cereals, cereal products, oilseeds, pulses, spices, dried fruits, cured tobacco leaves, and some animal products, and adult rice weevils cause serious damage to rice grains and to several cereal grains and beans [8]. Both are small insects with a body length of approximately three to four millimeters, but their behavior differs enough to provide useful variety from an ethological perspective.

The objective of this work was to construct a simple but unique experimental instrument consisting of an insulated conductor ring and a grounded metal pole designed to form an electric field in the space between them. The pole was designed for the insects to climb under different electrostatic conditions. Through this experiment, we investigated how the insects were able to sense the force generated by an electric field.

## 2. Materials and Methods

### 2.1. Insects

**Figure 1:**
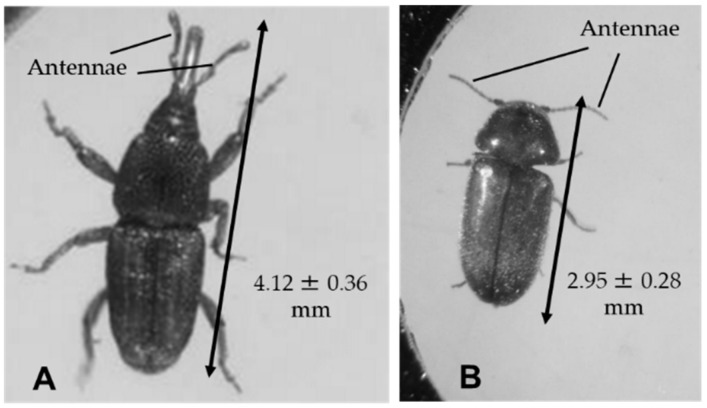
Body length of rice weevil (**A**) and cigarette beetle (**B**). The body length was measured using twenty adults of each insect species.

Rice weevil (*Sitophilus oryzae* Linnaeus, 1763) and cigarette beetle (*Lasioderma serricorne* Fabricius, 1792) adults were used for the pole-climbing assay described below. Both insects were purchased from Sumika Technoservice (Hyogo, Japan) and maintained in a growth chamber (25.0 ± 0.5°C, 12 h photoperiod at 4000 lux) using our standard method [7,9]. Newly emerged adult insects were used in subsequent experiments. The average body size of the adult rice weevils and cigarette beetles and their antennae are shown in Figure 1.

### 2.2. Electrostatic Instrument

**Figure 2:**
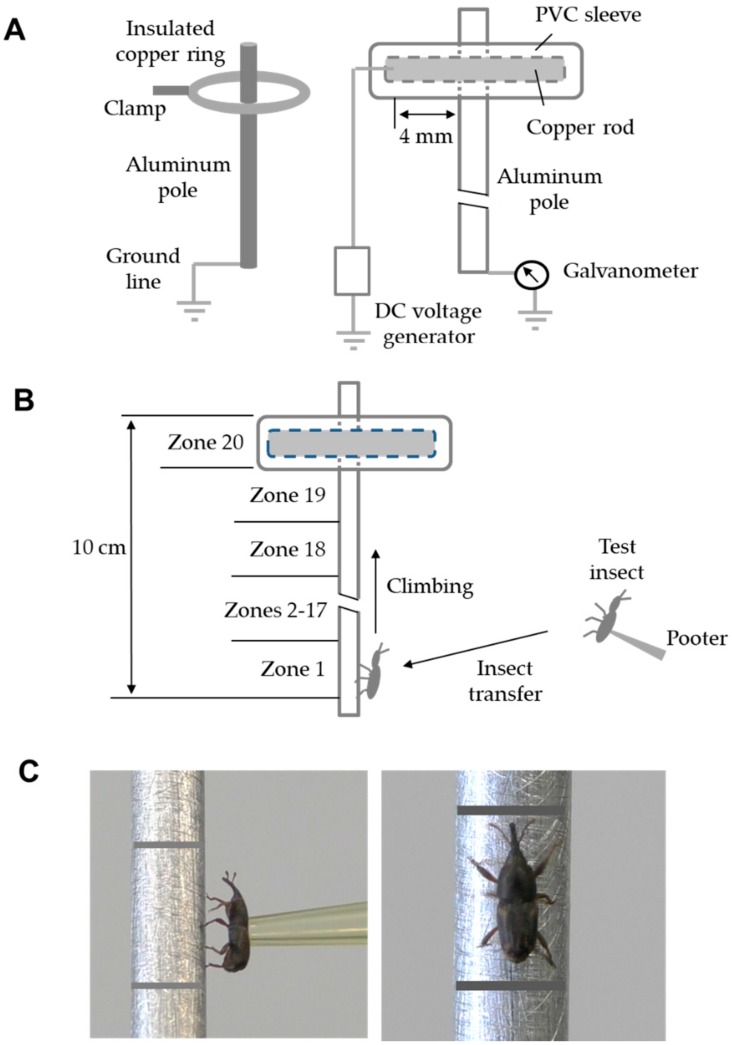
(**A**) Schematic representation of the electrostatic instrument used in the pole-climbing assay and its cross-sectional view, (**B**) insect transfer to zone 1 of a grounded aluminum pole, and (**C**) photographic demonstration of a rice weevil being held using the tip of the pooter (left) and then being placed on the aluminum pole (right). PVC, polyvinyl chloride.

The electrostatic instrument used for the pole-climbing assay is shown in Figure 2A. A copper ring (rod diameter: 2 mm; inner ring diameter: 10 mm) was insulated by passing it through a polyvinyl chloride (PVC) sleeve (10^9^ Ωcm) (Toalon, Tokyo, Japan) and holding horizontally with a clamp. The insulated conducting copper ring (ICR) was linked to a direct current (DC) voltage generator (Max Electronics, Tokyo, Japan) and negatively charged with different voltages (−1 to −15 kV). A straight aluminum rod (2 mm diameter, 250 mm length) intended as a pole for insects to climb was erected at the central position in the ICR and linked to a grounded line that was integrated with a galvanometer PC 720M PCLink7 (Sanwa, Tokyo, Japan) to measure the electricity that moved to the ground via the aluminum pole. The profiles of the electric currents were recorded with a current detector (detection limit, 0.01 µA) integrated into the galvanometer.

### 2.3. Pole-Climbing Assay

The instrument’s aluminum pole was marked with lines every 5 mm to create twenty zones, including the ICR region (zone 20) (Figure 2B). Adult insects were collected using an insect aspirator and placed at zone 1 (starting zone). The pooter, a long piece of flexible tubing with a plastic tip on one end, is a simple tool commonly used by entomologists in the field and lab to catch small insects; the tip is used to aspirate them into a tube and blow them out again at the desired location. Figure 2C shows the adult rice weevil captured with the tip of the pooter and placed on an aluminum pole.

In the first experiment, the rice weevils were placed in the starting zone of the aluminum pole and the ICR was charged by switching on the voltage generator immediately after the insects reached the designated zones (zones 2–20). First, we examined the voltages and zones that attracted insects to the ICR from the pole. In this experiment, negative voltages (−2 to −14 kV) were used to charge the ICR. At the same time, the transient release of negative electricity (free electrons) from attracted insects was detected by the galvanometer integrated in the grounded line as movement of insect discharge-mediated electricity to the ground.

In the second experiment, the ICR was charged with different voltages (−1 to −10 kV) when the rice weevil entered the zones beneath the insect attraction zones. Insect activity within the non-attraction zones was recorded on video to analyze avoidance behaviors. Twenty insects were used for each zone and each voltage.

In the third experiment (insect repulsion test), two insects (rice weevil and cigarette beetle) were used. The ICR was negatively charged with different voltages (−1 to −10 kV) and the insects were then placed in the starting zone on the aluminum pole. The pole-climbing insects were recorded on video to examine the positions where they stopped climbing and turned around. The climbing distance was determined by measuring the distance from the upper border of zone 1 to the parietal region of the insects’ heads when they stopped. Twenty insects from each species were used for each voltage.

All experiments were conducted in a room controlled at 25 °C and 40% relative humidity, using different insects for each replicate. The experiments were repeated five times; data are presented as means and standard deviations. The data were analyzed statistically, as described in the figure and table captions.

## 3. Results and Discussion

Insects are known to have distinctive responses to external physical and chemical stimuli [10]. Electric fields are a physical phenomenon that can have a strong influence on insect behavior. However, this phenomenon is puzzling, as insects do not encounter electric fields, especially high voltage-mediated electric fields produced in the present study, under natural conditions. Indeed, our previous study [7] was one of the first to directly examine interactions between insects and electric fields.

### 3.1. Electric Current Generation by Silent Discharge of ICR

The electrostatic instrument used in this study consisted of an insulated ring-shaped conductor and a grounded conductor pole placed vertically at the center of the ring. The insulated conductor was linked to a voltage generator to receive negative electricity, which dielectrically polarized the insulator covering the charged conductor; the outer surface of the insulator cover was negatively charged and the inner surface positively charged. The surface negative charge of the insulated conductor caused electrostatic induction of the grounded pole, forming an electric field between the insulated conductor rod and the grounded pole.

The flow of the accumulated electricity in the ICR depended on the voltage applied to the ICR, the electrode separation distance (distance between the ICR and grounded aluminum pole), the insulation resistance of the ICR cover sleeve, and the air conductivity between the two electrodes. The electric current from the insulated conductor was inversely proportional to the distance [1]. Moreover, the current depended on the insulation resistance at a given voltage, which determined the level of insulator conductivity [11]. Air conductivity changes in response to changes in water vapor concentration (relative humidity) of the air, becoming higher (i.e., greater amounts of electricity are transferred) under higher relative humidity [12]. In the present study, the voltage determined the current generation because all experiments were conducted under fixed instrumental and environmental conditions.

**Figure 3:**
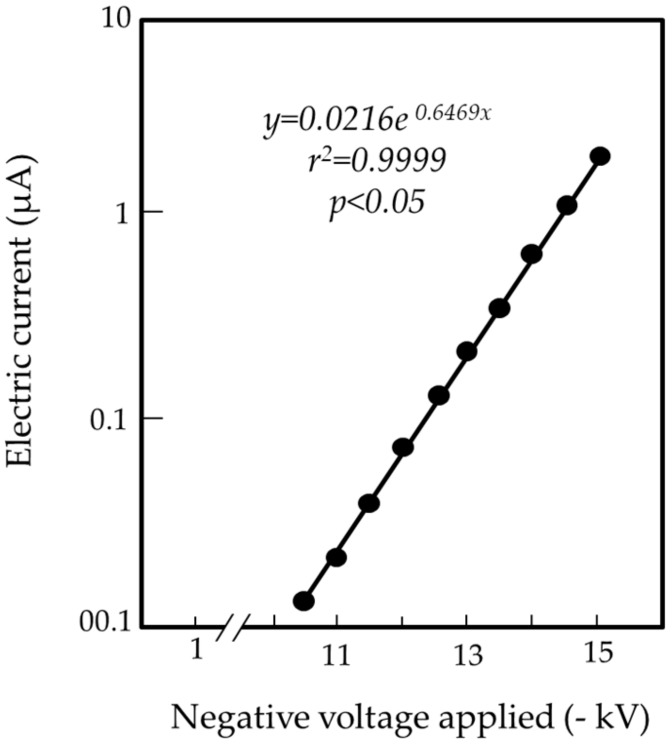
Relationship between the voltages applied to the insulated ring-shaped copper rod (ICR) and the current magnitudes produced in the electrostatic instrument. An exponential trend line was generated by plotting the points.

We examined the relationship between the electric current generated by the ICR and the voltage applied. Based on the instrument configuration, electricity accumulated in the ICR was transferred to the ground via the aluminum pole through mechanical discharge (silent discharge) from the ICR. This movement of electricity was recorded by the galvanometer integrated into the ground line. Figure 3 shows that the instrument generated an electric current at > −10 kV. The current was stable and its magnitude increased with increased applied voltage; indeed, there was a linear relationship between the applied voltage and the magnitude of the current generated.

### 3.2. Insect Discharge-Mediated Electric Current Generation for Insect Attraction

The most important characteristic of the electric field was the negative charge of the ICR. This negative charge created a strong repulsive force against other negatively charged entities (electrons) in the electric field, pushing them toward the ground via the grounded conductor. By this mechanism, any conductor that enters this field is deprived of its free electrons and becomes positively electrified (i.e., positively charged). This phenomenon has been defined as discharge-mediated positive electrification of the conductor [6,11].

The focus of the present study was on how insects responded to the electric field. Most insects possess a solid cuticle layer, the outer protective layer that covers the body of many invertebrates [13]. This layer is known to be conductive [14,15]; thus, an insect that enters the electric field is deprived of free electrons in the cuticle layer and becomes positively charged. This implies that discharge-mediated positive electrification can be induced in insects as the free electrons of the cuticle layer move to the earth. Positively electrified insects were attracted to the insulated conductor [2,3]. This capturing mechanism is applicable to nearly all insects, as most insects possess a conductive cuticle layer [6].

In the first pole-climbing experiment, we examined whether the instrument was capable of attracting insects. Rice weevils, which have the habit of climbing erect poles, were the most appropriate model insect for the present study. Indeed, they climbed to the top of the pole at a constant pace (6.4 ± 0.9 mm/sec, average of 10 insects) (Video S1A) when the ICR was not charged. The point of this experiment was to subject the insects climbing the pole to an attractive force. Video supplements show two typical instances of insects moving from the pole to the ICR; in one, the insects are attracted by the ICR and successfully caught (Video S1B and C), whereas in the other, the insects are attracted by the ICR and then sent flying away by a strong collision against it (Video S1D). The latter outcome was the more common (more than 90% incidence) in the present experiment. In both cases, it was clear that insects were exposed to an attractive force when they climbed the pole.

**Figure 4:**
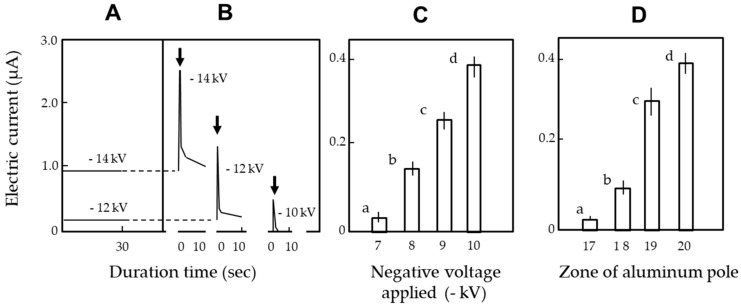
(**A**) Constant electric current by silent discharge of an insulated copper ring (ICR), (**B**) transient electric current from insect discharge (at −10 kV) or insect discharge plus constant discharge of ICR (at −12 and −14 kV), and (**C**) comparison of insect discharge-mediated transient electric currents in zone 20 at different voltages and (**D**) in different zones of an aluminum pole exposed to −10 kV-charged ICR. A: ICR was negatively charged with −10, −12, and −14 kV in the absence of insects on the pole. B: ICR was charged immediately after the insect entered the target zone. Arrow represents the timing of charging. C: ICR was negatively charged at −7 to −10 kV when the insect entered the target zone (zone 20). D: ICR was negatively charged at -10 kV when the insect entered the target zones. In C and D, twenty adult insects were used per experiment, and the means and standard deviations were calculated from five experimental replicates. The letters (a–d) on each column indicate significant differences (*p* < 0.05) according to Tukey’s method.

Figure 4A shows the transient electric current generation. At > −10 kV, the magnitude of the transient electric current was recorded as the sum of the constant silent discharge of the charged ICR. To avoid the effects of the silent discharge, subsequent assays were conducted in a voltage range of −1to −10 kV, which did not cause mechanical discharge. The magnitude of the transient electric current significantly differed among the voltages (Figure 4B) and zones tested (Figure 4C); higher voltages and zones resulted in transient electric current of larger magnitude. These results indicated that the charged ICR created an electrostatic force, depending on the voltage applied, and the force forced electricity (free electrons) out of the insect, positively polarizing the insect body. This polarization caused the insect to be attracted to the negatively charged ICR. Table 1 shows the percentage of insects attracted to the ICR, which was negatively charged at the designated voltage at the moment the insect entered the tested zone (target zone). Higher voltages resulted in stronger attractive force acting upon insects, recruiting them from more distant zones.

Incidentally, when the ground line was detached from the aluminum pole, the insects climbed the pole without stopping and passed through the charged ICR regardless of the voltage applied. The insects exhibited no avoidance behavior while climbing, and there was neither insect discharge-mediated transient electric current nor silent discharge from the ICR. These results suggest that the movement of electricity to the ground is necessary for insects to sense not only the strong force that attracts their bodies but also the weak one that affects their antennae. However, the movement of detectable amounts of electricity (i.e., detectable electric current) was limited in the former case, while in the latter case, the electric current was below the detection limit. A previous study that employed unipolar electric fields (where no electric current is present) found that an attractive force was generated as a result of uneven charge distribution in the antennae of the model insects (cockroaches); positive charges were attracted to the negatively charged electrodes [16]. The present non-grounded condition created a similar electric field with no electric current. However, this field did not result in avoidance behavior among insects.

A charged object (source charge) creates an electric field in the surrounding space. The strength of the electric field depends on the distance from another charged object (test charge) that enters this field; the strength of the electric field of the source charge can be measured by any other charge placed somewhere in its vicinity [17,18]. When placed within the electric field, the test charge will experience an electric force (either attractive or repulsive). In the instrument used in the present study, the electric field was formed in the space between the charged ICR and the grounded aluminum pole. The negatively charged ICR was the source charge and the positively polarized insect body was the test charge. The magnitude of the electric field strength is defined based on how it is measured [18]. In our approach, the electric field strength was defined as the repulsive force that drove free electrons out of the insect body, leading to positive electrification of the body. The attractive force was generated between the negatively charged ICR and positively charged insect body or body part. Based on our results, we concluded that insects can recognize electric field strength based on the attractive force imposed on their bodies.

### 3.3. Ethological Characterization of Insects Avoiding the Electric Field

The purpose of the second experiment was to assess insect behavior in zones where no insect movement toward the ICR was induced. Antennae are a specialized organ for sensing various external stimuli [10]. Newland et al. [16] reported that cockroaches detected electric fields (formed by a monoelectrically charged pole) with their antennae; cockroaches, when subjected to an electric field, deflected their antennae against the attraction forces, moving their antennae toward the electrode. Nonomura et al. [19] reported that whiteflies placed their antennae inside an electric field formed between opposite poles. This activity was similar to a “searching” behavior and the insects were deterred from entering the electric field. In the present study, special attention was paid to the perception of the electric field by the rice weevils’ antennae.

At a voltage of −10 kV, insect attraction occurred in the uppermost five zones (zones 16–20) (Table 1). In zones 14 and 15, the force was also strong; insects were deflected off center when the ICR was charged. However, insects immediately changed their direction of movement and turned downward (Video S1E). In zone 13, where the force became weaker, insects performed the distinctive behavior of vigorously and incessantly pulling at their antennae with their forelegs, seemingly to move the antennae back to the correct position (Video S1F). This behavior was also detected in zones 5–12, although it became more sluggish in these zones. In zones 5–15, after taking this action, the insects changed direction and descended the pole. Based on our understanding, the insects were subjected to an attractive force that pulled their antennae toward the ICR and the abovementioned behavior was likely aimed at opposing this force. In zones 1–4, the insect took no action when the ICR was charged and kept climbing, then returned when it reached the stopping–returning zone (zone 5). These results suggest that the antennae were used to recognize the weak attractive force of the electric field. More interestingly, the rice weevils seemed to regard this force as an external signal of risk or danger, abandoning their intrinsic habit of climbing the pole, instead returning back the way they came to avoid danger.

### 3.4. Pole-Ascending–Descending Behavior by Insects: A Simple Method to Examine Their Response to Electric Field Intensity

**Figure 5:**
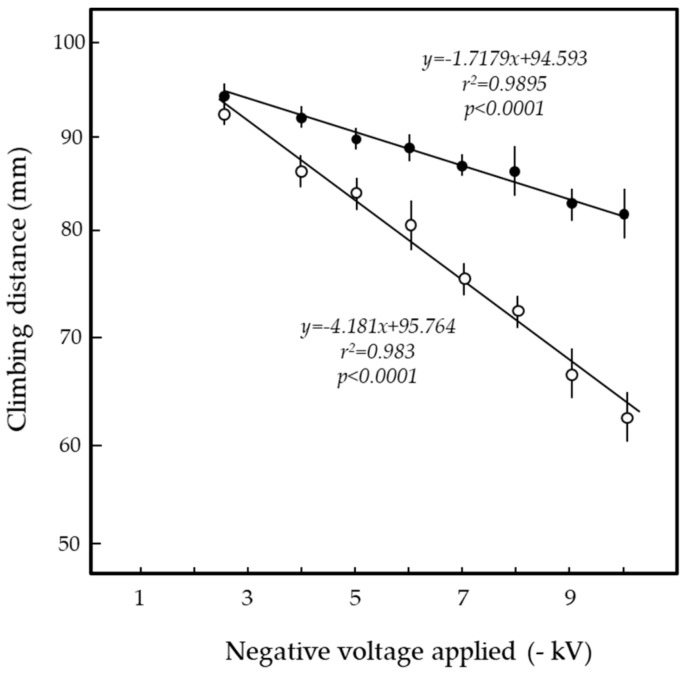
Pole-climbing behavior by rice weevils (closed circles) and cigarette beetles (open circles) placed in the electric field of the insulated ring-shaped rod (ICR) negatively charged with different voltages. A regression line was generated by plotting the points. Twenty adult insects were used per experiment, and the means and standard deviations were calculated from five experimental replicates.

In the third pole-climbing experiment (insect repulsion test), we determined the location of the stopping–returning point (SR point) for each voltage. The rice weevils responded changes in the electric field caused by changes in the voltage applied to the ICR. The location of the SR point moved higher on the pole with decreasing applied voltage (Figure 5 and Video S2A). The rice weevil’s pole-ascending–descending behavior was a simple and reliable ethology-based indicator to determine the presence of the attractive force generated by the electric field. Figure 5 shows the results for the cigarette beetle, which were consistent with those for the rice weevil; the cigarette beetles performed the same pole-ascending–descending behavior according to changes in the electric field due to the applied voltage (Video S2B). In summary, it appears that there is a clear reciprocal relationship between the distance that the insects climb up the grounded pole and the voltage applied to the ICR to create the electric field.

In comparing these two test insects, we identified a clear difference between them in force sensitivity; at all voltages, the cigarette beetle turned around in lower zones on the pole (Figure 5). It is likely that the longer antennae of the cigarette beetle (see Figure 1) were able to sense the force at a lower position on the pole than the rice weevil could. In our preliminary survey, some pole-climbing ladybug species (Asian ladybird beetle, 28-spotted ladybird beetle, and yellow ladybug) were deterred by the force of the electric field. Thus, the insects’ pole-ascending–descending behavior reflects their avoidance of a force recognized by their antennae. The major finding of the present study was that the pole-ascending–descending behavior was a reproducible and reliable biological indicator of the attractive force of the electric field (i.e., the field strength).

**Figure 6:**
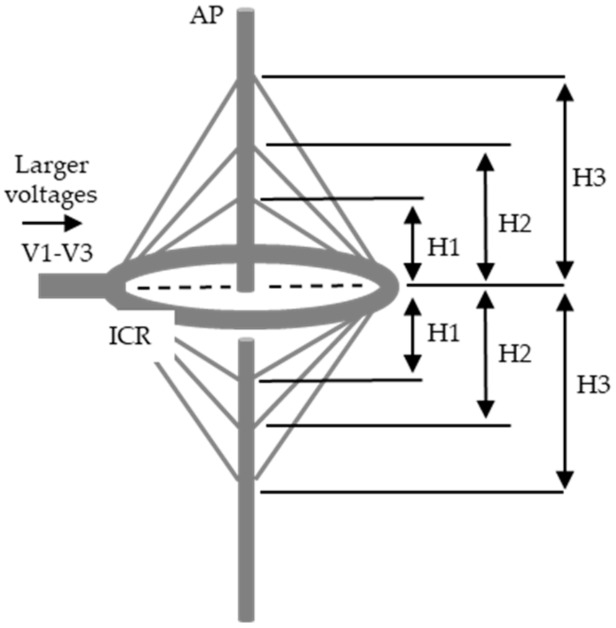
Conceptual representation of conical electric fields symmetrically formed above and below to form conic electric fields of different heights (H1–H3, corresponding to V1–V3, respectively). The height of the field was determined by the pole-ascending–descending (or pole-descending/ascending) action of the test insect.

One advantage of this experimental system was that regions of the pole both below and above the ICR could be used for the analysis of insect behavior. Under the non-charged condition, insects climbed to the top of the pole and remained there for 10–30 sec, then climbed down to the bottom of the pole. We exploited this behavior to investigate the formation of the electric field above the ICR. Specifically, we charged the ICR when the insects began descending from the top of the pole. As expected, the insect stopped at the position corresponding to the edge of the electric field and turned around to ascend the pole again (this behavior is referred to as the pole-descending/ascending action). Based on these results, we inferred the formation of symmetric conical electric fields above and below the ICR (Figure 6).

## 4. Conclusions

The electrostatic instrument devised in the present study has a simple but unique configuration that enables electrostatic-based entomological research. This study was based on the knowledge that the model insects have a habit of climbing erect poles and that the insects could be subjected to an electric field while climbing. The insects performed this behavior very consistently and their responses to the imposed force were, therefore, highly reproducible. The insects could recognize the existence of the electric field by the force imposed on their antennae. Sensing the electric field caused the insects to stop climbing and quickly retrace their steps. Apparently, the external force was interpreted as a potentially risky signal, such that the insects undertook danger avoidance behavior. This response resulted in the observed pole-ascending–descending action. The insects were highly responsive to changes in the electric field. Many species of insects exhibit pole-climbing behavior; therefore, this system may be used to compare their ability to sense electric fields. Thus, the present work provides an experimental basis for ethological research on insect responses to electric fields.

## Figures and Tables

**Table 1:** Percentage of adult rice weevils attracted to the insulated ring-shaped copper rod (ICR), which was negatively charged with different voltages.

Target Zones for Charging	Negative Voltage (- kV) Applied to ICR							
	3	4	5	6	7	8	9	10
15	0	0	0	0	0	0	0	0
16	0	0	0	0	0	0	2.0 ± 2.7 a	13.0 ± 5.7 a
17	0	0	0	0	6.0 ± 5.5 a	14.0 ± 4.2 a	23.0 ± 5.7 b	35.0 ± 7.9 b
18	0	0	5.0 ± 3.5 a	19.0 ± 4.2 a	22.0 ± 5.7 b	37.0 ± 6.7 b	59.0 ± 7.4 c	96.0 ± 4.2 c
19	0	7.0 ± 5.2 a	19.0 ± 4.2 b	32.0 ± 2.7 b	52.0 ± 2.7 c	100 c	100 d	100 d
20	19.0 ± 4.2	39.0 ± 6.5 b	69.0 ± 4.2 c	100 c	100 d	100 c	100 d	100 d

The ICR was charged when the insect entered the tested zone. Twenty insects were used for each voltage and zone, and the means and standard deviations were calculated from five experimental replicates. Letters (a–d) indicate significant differences within each vertical column (*p* < 0.05) according to Tukey’s method.

## Supplementary Materials

The following are available online at https://www.mdpi.com/2075-4450/11/3/187/s1. Video S1: (A) Climbing an erect pole by test insect (adult rice weevil), (B) ICR-attraction and catch of test insect in zone 20, (C) in zone 18, (D) collision to ICR by attracted test insect, (E) deflection and U-turn by test insect, and (F) touch to antennae with insect’s forelegs. Video S2: Pole-ascending–descending behavior by adult rice weevil (A) and cigarette beetle (B) at -3, -6, and -9 kV (from left to right) applied to ICR.
